# Increased Risk of Chronic Kidney Disease in Rheumatoid Arthritis Associated with Cardiovascular Complications – A National Population-Based Cohort Study

**DOI:** 10.1371/journal.pone.0136508

**Published:** 2015-09-25

**Authors:** Hsien-Yi Chiu, Hui-Ling Huang, Chien-Hsun Li, Hung-An Chen, Chia-Lun Yeh, Shih-Hsiang Chiu, Wei-Chun Lin, Yu-Pin Cheng, Tsen-Fang Tsai, Shinn-Ying Ho

**Affiliations:** 1 Institute of Biomedical Engineering, College of Medicine and College of Engineering, National Taiwan University, Taipei, Taiwan; 2 Department of Dermatology, National Taiwan University Hospital Hsin-Chu Branch, Hsinchu, Taiwan; 3 Department of Dermatology, National Taiwan University Hospital and National Taiwan University College of Medicine, Taipei, Taiwan; 4 School of Medicine, Fu Jen Catholic University, New Taipei City, Taiwan; 5 Institute of Bioinformatics and Systems Biology, National Chiao Tung University, Hsinchu, Taiwan; 6 Department of Biological Science and Technology, National Chiao Tung University, Hsinchu, Taiwan; 7 Division of Neurosurgery, Department of Surgery, National Taiwan University Hospital Hsin-Chu Branch, Hsinchu, Taiwan; 8 Department of Dermatology, Cathay General Hospital, Taipei, Taiwan; University of Sao Paulo Medical School, BRAZIL

## Abstract

**Background and Objectives:**

There have been few large population-based studies of the association between rheumatoid arthritis (RA) and chronic kidney disease (CKD) and glomerulonephritis. This nationwide cohort study investigated the risks of developing CKD and glomerulonephritis in patients with RA, and the associated risks for cardiovascular complications.

**Methods:**

From the Taiwan National Health Insurance Research Database, we identified a study cohort of 12,579 patients with RA and randomly selected 37,737 subjects without RA as a control cohort. Each subject was individually followed for up for 5 years, and the risk of CKD was analyzed using Cox proportional hazards regression models.

**Results:**

During the follow-up period, after adjusting for traditional cardiovascular risk factors RA was independently associated with a significantly increased risk of CKD (adjusted hazard ratio [aHR] 1.31; 95% confidence interval [CI] 1.23–1.40) and glomerulonephritis (aHR 1.55; 95% CI 1.37–1.76). Increased risk of CKD was also associated with the use of non-steroidal anti-inflammatory drugs, cyclosporine, glucocorticoids, mycophenolate mofetil, and cyclophosphamide. Patients with comorbidities had even greater increased risk of CKD. Moreover, RA patients with concurrent CKD had significantly higher likelihood of developing ischemic heart disease and stroke.

**Conclusions:**

RA patients had higher risk of developing CKD and glomerulonephritis, independent of traditional cardiovascular risk factors. Their increased risk of CKD may be attributed to glomerulonephritis, chronic inflammation, comorbidities, and renal toxicity of antirheumatic drugs. Careful monitoring of renal function in RA patients and tight control of their comorbid diseases and cardiovascular risk factors are warranted.

## Introduction

Rheumatoid arthritis (RA) is a chronic inflammatory autoimmune disease that affects many body tissues and leads to joint destruction and other major morbidity and mortality. In particular, previous reports have indicated that patients with RA also have considerable incidence of renal disease. Specifically, there is accruing evidence that a substantial proportion of patients with early RA have proteinuria, hematuria and renal dysfunction [1,2].

Renal disease in RA is clinically important because it not only restricts the management of primary disease, but also increases mortality. In one study, RA patients with renal disease had significantly increased mortality compared to those with normal renal function, with a hazard ratio (HR) of 2.77–4.45 [3]. Other investigators have shown that subjects hospitalized for RA were significantly more likely to die from renal failure than the general population: HR 3.1 (95% confidence interval [CI]: 2.5–3.9) for males, and HR 3.5 (95% CI: 3.0–4.0) for females [2,4]. Autopsy findings in patients with RA have shown that renal failure is a major cause of death in 3–20% of cases [5,6].

However, previous studies evaluated a variety of kidney disorders or used different criteria to define renal abnormality in RA. Further major limitations were small sample sizes, cross-sectional design, sampling frame at consecutive times from a single rheumatology clinic, lack of a comparison group, and short follow-up, all of which make it difficult to determine the true magnitude of risk and potentially limit the generalizability of their results. Few studies have specifically investigated the chronic kidney disease (CKD) in patients with RA and despite evidence that Asians have higher prevalence of CKD than Caucasians, such research focusing on Asian subjects is lacking [7]. Furthermore, as treatment patterns for RA have changed over the years, the true incidence of CKD may be different nowadays and remains unclear. This study was conducted to determine the risk of CKD and glomerulonephritis (GN) and the associated risk for cardiovascular (CV) complications in a nationally-representative cohort of patients with RA from Taiwan.

## Materials and Methods

### Dataset

This retrospective cohort study used data from the Taiwan Longitudinal Health Insurance Database (LHID) 2005, which is a subset of the National Health Insurance Research Database (NHIRD). NHIRD data are compiled from the Taiwan National Health Insurance (NHI) system, which was launched in 1995 to finance health care for all citizens and provides care for approximately 99% of the Taiwanese population of more than 23 million. In the LHID 2005, approximately 1,000,000 representative individuals were randomly sampled from among all of those in NHIRD that year. The database includes inpatient care, outpatient care, ambulatory care and prescription drugs from 1 January 2000 through 31 December 2010. A multistage stratified systematic sampling design was used and there were no statistically significant differences in gender, age, or average insured payroll-related amount, between the LHID sample and the whole NHIRD population [8]. The disease diagnoses used in our study were coded using the International Classification of Diseases, 9^th^ Revision, Clinical Modification (ICD-9-CM). The local Investigational Research Bureau approved the study (HCH 103-024-E).

### Study Population

The RA study cohort comprised 12,579 patients newly diagnosed with RA (ICD-9-CM 714.0, 714.2, and 720.0) between 1 January 2001, and 31 December 2005. The initial diagnosis date was defined as the index date of entry into the RA cohort. Patients who were younger than 18 or who had CKD within 1 year before the index date were excluded. A control cohort of 37,737 subjects who had not been diagnosed with RA from year 2000 through 2010, were selected to match each RA patient for gender and age.

### Study Outcomes

**Fig 1:**
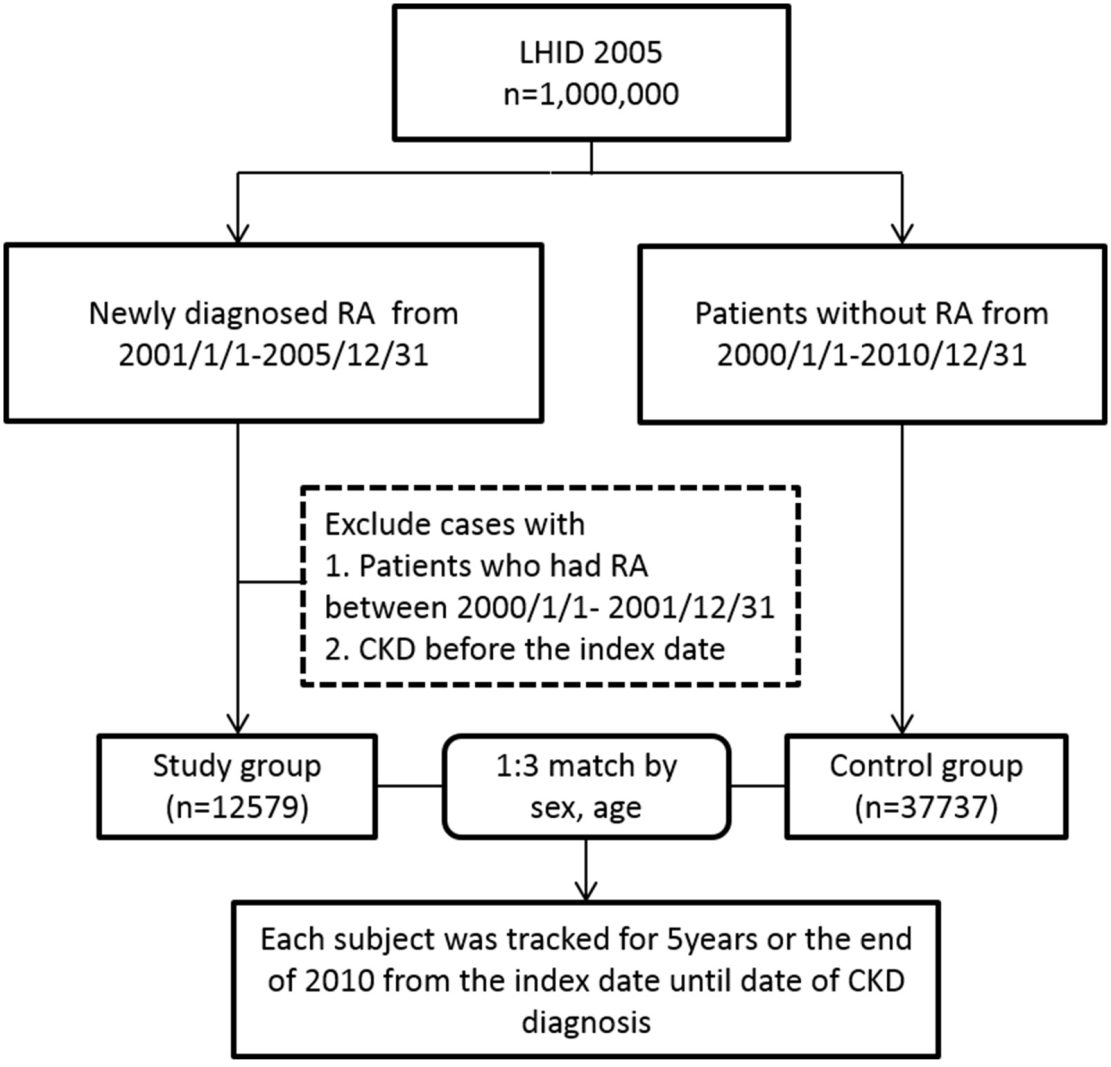
Study cohort. Subject flow for study cohort.

The primary study outcomes were new-onset CKD (ICD-9-CM 580, 581, 582, 583, 584, 585, 586, 587, 588, 589, 753, 403, 404, 2504, 2741, 4401, 4421, 4473, 5724, 6421, 6462) and GN (ICD-9-CM codes: 580.0, 580.1, 580.2, 580.3, 580.4, 580.9, 590.81, 582.0, 582.1, 582.4, 582.81, 582.89, 582.9, 583.0, 583.1, 583.2, 583.4, and 583.9); final-stage CKD (end-stage renal disease [ESRD]) (ICD-9-CM 585) was the secondary outcome. Patients were followed-up for 5 years from the index date or until the development of CKD (Fig 1). Comorbidities included diabetes mellitus (ICD-9-CM 250.xx), hypertension (ICD-9-CM 362.11, 401–405, and 437.2), hyperlipidemia (ICD-9-CM 272.x), CV disease (ICD-9-CM 410–429), and obesity (ICD-9-CM 278.0x).

### Statistical Analysis

SPSS software version 19.0 (SPSS Inc., Chicago, IL, USA) was used for all statistical analyses. A two-sided p-value <0.05 determined statistical significance. Microsoft SQL Server 2008 software was used for data management and analysis.

Pearson’s chi-square was used to compare the distributions of demographic characteristics between patients with and without RA, and for evaluating differences between categorical variables. Cox proportional hazard regression was performed to estimate the HR and 95% CI of RA associated with CKD. The covariate-adjusted HR was analyzed after adjusting for significant factors (p-value <0.05).

## Results

Table 1 shows the baseline distributions of demographic characteristics and clinical features, such as comorbidities and medication use, that occurred throughout the study period among patients with and without RA. There were no significant differences between RA patients and controls in gender or age. However, several comorbidities were significantly more prevalent among RA patients than controls; these included diabetes, hypertension, hyperlipidemia, CV disease, and obesity (all p <0.001). Compared with RA patients, a significantly lower proportion of controls were prescribed glucocorticoids, disease modifying antirheumatic drugs (DMARDs), non-steroidal anti-inflammatory drugs (NSAIDs) and biologics; however, some control patients also received short-term and infrequent glucocorticoids or NSAIDs for common ailments, such as upper respiratory infections, lower back pain and skin allergy (S1 Table).

**Table 1:** Gender, age, urbanization, geography comorbidity, and medications distributions among individuals with and without rheumatoid arthritis.

Variable	Numbers (proportions) of individuals		P-value
	Patients with RA(N = 12579)	Patients without RA(N = 37737)	
**Sex**			1.00
Female	7580 (60.3%)	22740 (60.3%)	
Male	4999 (39.7%)	14997 (39.7%)	
**Age** (years)			1
18–29	1693 (13.5%)	5079 (13.5%)	
30–39	1982 (15.8%)	5946 (15.8%)	
40–49	2889 (23.0%)	8667 (23.0%)	
50–59	2535 (20.2%)	7605 (20.2%)	
60–69	1901 (15.1%)	5703 (15.1%)	
≥70	1579 (12.6%)	4737 (12.6%)	
**Income** ^a^			<0.001
<18,000	5269 (41.9%)	16923 (44.8%)	
18,000–34,000	5465 (43.4%)	15567 (41.3%)	
≥35,000	1845 (14.7%)	5247 (13.9%)	
**Urbanization**			0.22
Provinces	3232 (25.7%)	10024 (26.6%)	
Counties	998 (7.9%)	2975 (7.9%)	
Districts	3255 (25.9%)	9770 (25.9%)	
Urban villages	5094 (40.5%)	14968 (39.7%)	
**Geography**			<0.001
North	6510 (51.8%)	18787 (49.8%)	
Central	2161 (17.2%)	6686 (17.7%)	
South	3488 (27.7%)	11357 (30.1%)	
East	420 (3.3%)	907 (2.4%)	
**Comorbidity**			
Hypertension	4756 (37.8%)	12197 (32.3%)	<0.001
Diabetes	2441 (19.4%)	5863 (15.5%)	<0.001
Cardiovascular disease	3999 (31.8%)	8581 (22.7%)	<0.001
Hyperlipidemia	3683 (29.3%)	8530 (22.6%)	<0.001
Obesity	166 (1.3%)	296 (0.8%)	<0.001
**Drug**			
Glucocorticoids	6055 (48.1%)	10639 (28.2%)	<0.001
Methotrexate	615 (4.9%)	74 (0.2%)	<0.001
Cyclosporine	99 (0.8%)	13 (0.0%)	<0.001
Azathioprine	105 (0.8%)	29 (0.1%)	<0.001
Mycophenolate Mofetil	7 (0.1%)	7 (0.0%)	<0.05
Hydroxyurea	3 (0.0%)	15 (0.0%)	0.41
Cyclophosphamide	49 (0.4%)	90 (0.2%)	<0.05
Sulfasalazine	1453 (11.6%)	81 (0.2%)	<0.001
Leflunomide	53 (0.4%)	4 (0.0%)	<0.001
Penicillamine	34 (0.3%)	8 (0.0%)	<0.001
Hydroxychloroquine	1181 (9.4%)	160 (0.4%)	<0.001
NSAIDs	12007 (95.5%)	28583 (75.7%)	<0.001
Etanercept	33 (0.3%)	1 (0.0%)	<0.001
Adalimumab	12 (0.1%)	0 (0.0%)	<0.001

NSAIDs, non-steroidal anti-inflammatory drugs; RA, rheumatoid arthritis.

^a^ New Taiwan dollars

**Fig 2:**
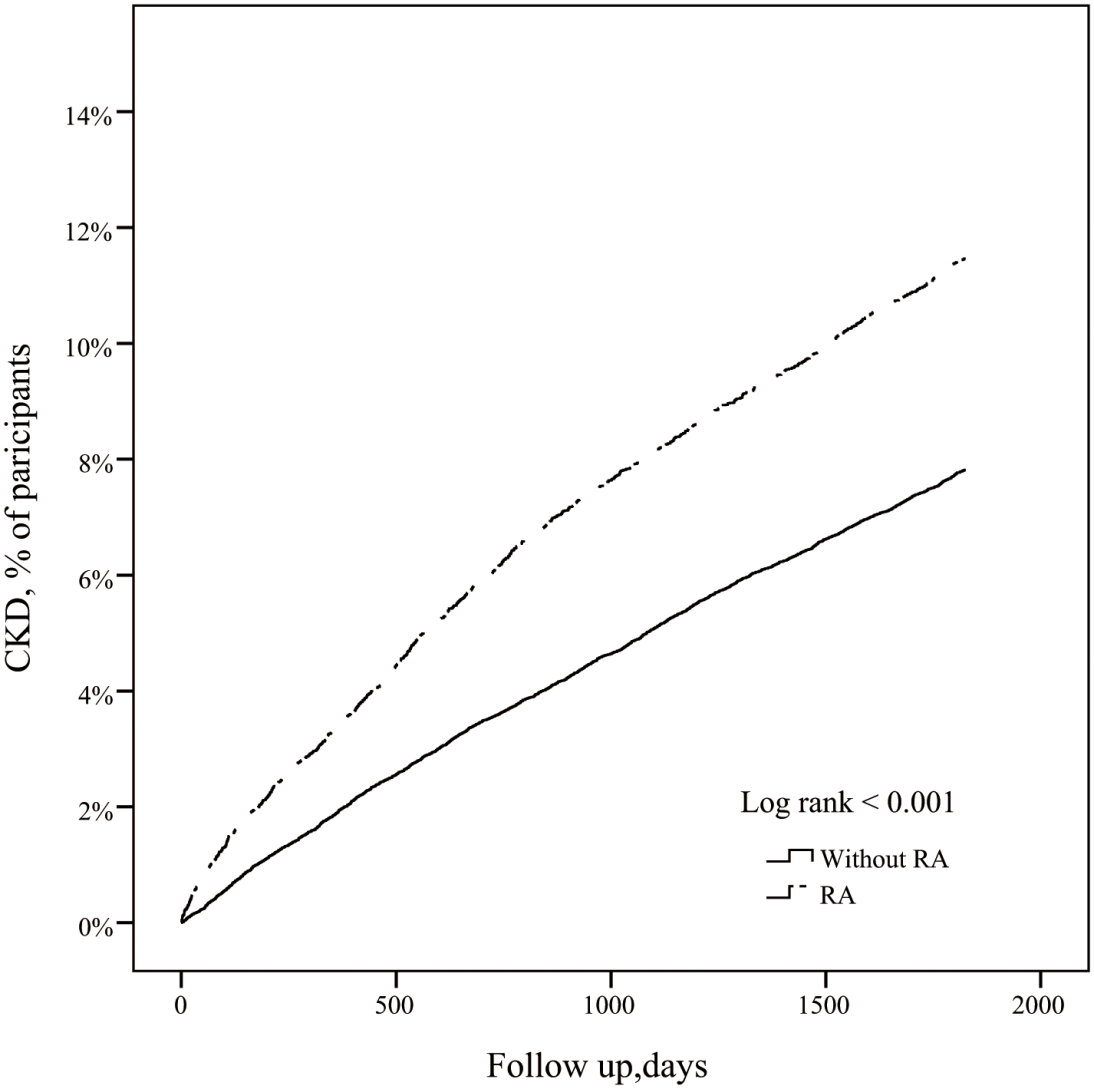
Kaplan-Meier survival curves. Kaplan-Meier survival curves for time to occurrence of chronic kidney disease among patients with and without rheumatoid arthritis.

Table 2 shows the incidence and HRs of CKD, GN and ESRD among patients with RA and without. Of 12,759 patients with RA, 1442 (11.5%), 166 (1.3%) and 379 (3.0%) patients respectively, developed CKD, ESRD and GN during the 5 year follow-up period. After adjusting for monthly income, age, gender, urbanization, medication, geography, and comorbidities, the adjusted HR (aHR) among patients with RA versus controls was 1.31 (95% CI 1.23–1.40, p <0.001) for CKD, 1.08 (95% CI 0.90–1.29, p = 0.43) for final- stage CKD (ESRD) and 1.55 (95% CI, 1.37–1.76, p <0.001) for GN. Fig 2 shows Kaplan-Meier survival curves for time to CKD occurrence for RA versus controls. Stratified by age, the aHRs for CKD, GN and ESRD increased with age and were highest in the oldest adults among both RA patients and controls. Moreover, the aHRs for CKD were higher in subjects with comorbidities than those without.

**Table 2:** Incidence rates and hazard ratios for CKD, ESRD, and GN in patients with rheumatoid arthritis.

Variable	CKD		ESRD		GN	
	Crude HR (95% CI)	Adjusted HR* (95% CI)	Crude HR (95% CI)	Adjusted HR* (95% CI)	Crude HR (95% CI)	Adjusted HR* (95% CI)
**Rheumatoid arthritis**	1.50 (1.41–1.60)^‡^	1.31 (1.23 to 1.4)^‡^	1.22 (1.02–1.46)^†^	1.08(0.90–1.29)	1.74 (1.53–1.974)^‡^	1.55(1.37–1.76)^‡^
**Incidence rate** ^a^	18.34		2.44		4.38	
**Gender**						
Female	1.00	ND	1.00	1.00	1.00	1.00
Male	1.01 (0.95–1.07)	ND	1.23 (1.05–1.45)^†^	1.47(1.25–1.74)^‡^	0.87 (0.77–0.99)^†^	1.01(0.89–1.15)
**Age** (years)						
18–29	1.00	1.00	1.00	1.00	1.00	1.00
30–39	1.45 (1.19–1.76)^‡^	1.27(1.05–1.54)^†^	1.85 (0.96–3.58)	1.75(0.91–3.40)	1.24 (0.87–1.77)	1.15(0.80–1.63)
40–49	2.44 (2.06–2.89)^‡^	1.57(1.32–1.87)^‡^	3.03 (1.67–5.5)^‡^	2.23(1.22–4.07)^‡^	2.08 (1.52–2.82)^‡^	1.55(1.13–2.12)^†^
50–59	4.09 (3.47–4.82)^‡^	1.83(1.54–2.17)^‡^	5.82 (3.27–10.34)^‡^	3.15(1.74–5.69)^‡^	3.10 (2.30–4.19)^‡^	1.76(1.29–2.42)^‡^
60–69	6.24 (5.3–7.35)^‡^	2.12(1.78–2.52)^‡^	11.1 (6.3–19.56)^‡^	4.49(2.48–8.10)^‡^	4.45 (3.30–6.00)^‡^	2.03(1.47–2.80)^‡^
≥70	8.8 (7.48–10.36)^‡^	2.82(2.37–3.37)^‡^	19.6 (11.2–34.31)^‡^	7.08(3.93–12.75)^‡^	5.82 (4.32–7.83)^‡^	2.53(1.83–3.50)^‡^
**Comorbidity**						
Hypertension	4.37 (4.1–4.66)^‡^	1.81(1.68–1.96)^‡^	5.11 (4.28–6.11)^‡^	1.73(1.40–2.15)^‡^	3.02 (2.67–3.42)^‡^	1.34(1.15–1.56)^‡^
Diabetes	4.23 (3.98–4.49)^‡^	2.04(1.91–2.18)^‡^	4.57 (3.88–5.38)^‡^	2.16(1.81–2.59)^‡^	2.86 (2.52–3.26)^‡^	1.47(1.28–1.69)^‡^
Cardiovascular disease	3.51 (3.31–3.72)^‡^	1.60(1.50–1.71)^‡^	4.1 (3.48–4.83)^‡^	1.70(1.42–2.05)^‡^	3.19 (2.83–3.60)^‡^	1.74(1.52–2.00)^‡^
Hyperlipidemia	3.3 (3.11–3.5)^‡^	1.57(1.47–1.68)^‡^	2.82 (2.39–3.31)^‡^	1.28(1.07–1.53)^‡^	2.87 (2.54–3.23)^‡^	1.63(1.42–1.87)^‡^
Obesity	1.81 (1.43–2.3)^‡^	1.16(0.91–1.47)	1.76 (0.91–3.39)^†^	1.39(0.71–2.69)^‡^	1.40 (0.81–2.43)	N/A

* Each variable was adjusted for every other variable listed for which crude HR was significant (p <0.05), and also for income

^†^ p<0.05 for comparison between patients with versus without rheumatoid arthritis.

^‡^ p<0.001 for comparison between patients with versus without rheumatoid arthritis.

^a^ Incidence rate: per 1000 person-years.

CI, confidence interval; CKD, chronic kidney disease; ESRD, end-stage renal disease; GN, glomerulonephritis; HR, hazard ratio; N/A, not applicable; NSAID, non-steroidal anti-inflammatory drug; OR, odds ratio; ND, not done.

Table 3 shows the risks of CKD among RA patients treated with various medications. Adjusted for gender, age, comorbidities, and every other drug listed that showed significant crude HR, patients who received glucocorticoids (p<0.001), mycophenolate mofetil (p<0.05), cyclophosphamide (p<0.05) or NSAIDs (p<0.05) had significantly increased risk of CKD. The overall OR for risk of CKD conferred by DMARDs was 1.22 (p<0.05). Moreover, frequent NSAID users had significantly higher likelihood of developing CKD than infrequent users and non-users.

**Table 3:** Risks of CKD among rheumatoid arthritis patients treated with medications.

Medication*	RA with CKD	RA without CKD	Crude OR (95% CI)	Adjusted OR**(95% CI)
**Glucocorticoids**	888	5167	1.85 (1.66–2.07)^‡^	1.49 (1.32–1.68)^‡^
Non-users	554	5967	1	1.00 (ND)
Infrequent users^a^	650	4137	1.64 (1.47–1.84)^‡^	1.40 (1.25–1.57)^‡^
Frequent users^b^	238	1033	2.33 (2.01–2.72)^‡^	1.75 (1.47–2.07)^‡^
**DMARDs**	264	1924	1.07 (0.93–1.24)	1.22 (1.02–1.45)^†^
Methotrexate	73	542	1.04 (0.81–1.34)^†^	1.07 (0.82–1.41)
Azathioprine	20	85	2.81 (1.48–5.30)^†^	1.74 (0.99–3.05)
Mycophenolate mofetil	4	3	1.83 (1.12–2.99)^†^	10.58 (2.13–52.54)^†^
Hydroxyurea	2	1	10.32 (2.31–46.17)^†^	13.16 (0.96–180.65)
Cyclophosphamide	13	36	15.47 (1.40–170.68)	2.57 (1.27–5.20)^†^
Sulfasalazine	164	1289	0.98 (0.83–1.17)	1.23 (0.99–1.51)
Leflunomide	10	43	1.80 (0.90–3.59)	1.87 (0.88–399.00)
Penicillamine	5	29	1.33 (0.52–3.45)	0.97 (0.35–2.65)
Hydroxychloroquine	155	1026	1.19 (0.99–1.42)	1.15 (0.93–1.43)
Gold	0	0	N/A	N/A
Cyclosporine	17	82	1.61 (0.95–2.72)	1.54 (0.84–2.82)
Non-users	1425	11055	1.00	1.00 (ND)
Infrequent users^a^	7	19	2.86 (1.20–6.81)^†^	3.01(1.18–7.71)^†^
Frequent users^b^	10	63	1.23 (0.63–2.41)	1.09 (0.51–2.35)
**NSAIDs**	1418	10589	3.06 (2.02–4.62)^‡^	1.68 (1.10–2.56)^†^
Non-users	24	548	1.00	1.00 (ND)
Infrequent users^a^	444	5678	1.79 (1.17–2.72)^†^	1.45 (0.95–2.22)
Frequent users^b^	974	4911	4.53 (2.99–6.85)^‡^	2.01 (1.31–3.09)^‡^
**Biologics**	5	40	0.97 (0.38–2.45)	0.86 (0.32–2.34)

^†^ p <0.05 for comparison between patients with rheumatoid arthritis and without rheumatoid arthritis.

^‡^ p <0.001 for comparison between patients with rheumatoid arthritis and without rheumatoid arthritis.

* Rheumatoid arthritis patients with one or more drug prescription during 5-year follow-up

** Adjusted for gender, age group, comorbidities and every other listed drug for which crude hazard ratio was significant (p <0.05).

CI, confidence interval; CKD, chronic kidney disease; N/A, not applicable; NSAID, non-steroidal anti-inflammatory drug; OR, odds ratio; RA, rheumatoid arthritis; ND, not done.

^a^ Prescribed <90 days.

^b^ Prescribed ≥90 days.

Table 4 shows the comparative risks of CV complications in RA patients with and without CKD. Adjusted for gender, age, diabetes, hypertension, and hyperlipidemia, RA patients with versus without CKD had significantly increased risks of ischemic heart disease (p<0.001) and stroke (p<0.05).

**Table 4:** The risk of cardiovascular complications in rheumatoid arthritis patients with versus without CKD.

Variable	RA with CKD	RA without CKD	Crude HR	Adjusted HR*
Ischemic heart disease	590	2119	2.95 (2.63–3.31)^‡^	1.57 (1.38–1.79)^‡^
Stroke	351	1274	2.49 (2.18–2.85)^‡^	1.24 (1.06–1.43)^†^

^†^ p <0.05 for comparison between rheumatoid arthritis patients with versus without.

^‡^ p <0.001 for comparison between rheumatoid arthritis patients with versus without.

*Adjusted for gender, age group, diabetes, hypertension, and hyperlipidemia.

CI, confidence interval; CKD, chronic kidney disease; HR, hazard ratio; RA, Rheumatoid arthritis.

## Discussion

This national population-based cohort study of more than 12, 000 RA patients who were followed for up to 5 years, not only affords generalizability in comparing the incidence of CKD in patients with versus without RA, but also makes it possible to assess the temporal relationship between RA and CKD.

The reported prevalence of kidney disease in patients with RA ranges from 5–50%, reflecting wide variations in the diagnostic criteria and definitions of renal disease, and different study designs [1,5,9,10]. In a cross-sectional population-based cohort study of 604 Finnish patients with RA, 17% had evidence of nephropathy (defined as hematuria, proteinuria, or kidney failure) [11]. The prevalence of kidney disease among 129 consecutive RA patients in a more recent study was 46.3% as measured by the Modification of Diet in Renal Disease formula, and 57% according to Cockcroft-Gault formula [2]. Another recent study of 350 consecutive RA patients in England found that 53% had mild renal impairment, with glomerular filtration rate (GFR) of 60–90 mL/min/1.73 m^2^, and 13% had moderate renal impairment, with GFR below 60 mL/min/1.73m^2^ [12]. Among another 400 RA patients, 11.5% had GFR below 60, and only four had severe renal impairment (GFR <30) [13]. However, specific data on the incidence of CKD in Asian patients with RA are lacking.

The cause of renal disease in RA patients is contentious, and may be attributable to nephrotoxic pharmacotherapies, secondary renal diseases induced by amyloidosis and/or GN, and associated comorbidities [5,13]. The etiologic role of chronic inflammation has also been highlighted [14,15,16,17]. Accumulating data reveal that CKD is more prevalent in patients with chronic inflammatory disorders, such as psoriasis [17,18] and ankylosing spondylitis [19], compared to the general population. Systemic inflammation may contribute to progressive loss of kidney function and anti-inflammatory drugs, such as TNF-α antagonists, have therapeutic potential in preventing CKD progression in RA [20,21,22]. Concerning comorbidities in CKD, Daoussis and coworkers showed renal dysfunction to be strongly associated with classic CV risk factors [13]. Likewise, our analysis found that diabetes, hypertension, hyperlipidemia, and CV disease were associated with the development of CKD in RA. Moreover, our results demonstrate that RA is associated with increased risk of CKD independently of traditional CV risk factors. In a recent study, excess weight rather than hypertension and diabetes appeared to be more strongly associated with CKD in RA [9]; however, we did not find a significant association between obesity and CKD after adjustment, probably due to ethnic differences or relatively lower prevalence of obesity in Asian populations.

Previous reports suggested that RA may be complicated by renal disease secondary to GN. Renal biopsies in 158 Japanese and 110 Finnish RA patients with clinical renal diseases, suggested that mesangial GN is the most frequent type of GN (34–36%), followed by membranous GN [23,24]. Other types of GN, including rapidly progressive GN, minimal change glomerulopathy, and immunoglobulin A nephropathy have also been reported in RA [1,2,23,24]. Since GN may progress to CKD and CKD is diagnosed based on abnormal urinalysis and GFR results, irrespective of the specific cause, we further investigated the relationship among GN, ESRD and RA. Our patients with RA were at higher risk of both GN and ESRD than controls. The increased risk of CKD may be partly attributed to higher incidence of GN in patients with RA.

Anti-inflammatory drugs have also been implicated in causing renal disease in RA patients [1,9,25,26]. Likewise, we found that use of NSAIDs was associated with the development of CKD in patients with RA in a dose-dependent manner. Also consistent with earlier studies, cyclosporine [26,27] and cyclophosphamide [28,29] were associated with nephrotoxicity that would lead to CKD in patients with RA. However, our data showed a higher likelihood of developing CKD in infrequent versus frequent cyclosporine users. Further analysis showed that infrequent users are older than frequent users, and the probable explanation for our result is that physicians may avoid prescribing frequent cyclosporine in elderly patients with RA who tend to be more likely to progress to CKD (S2 Table). Our observation that glucocorticoids, mycophenolate mofetil and cyclophosphamide were associated with increased risk of CKD might be because prescription of these drugs indicates higher disease activity or presence of comorbidity, resulting in rapidly declining renal function. Similarly, Hickson and coworkers found corticosteroids to be associated with increased risk of estimated GFR (eGFR) <45 mL/min/1.73 m^2^ in RA [9]. Nevertheless, the small sample size in the mycophenolate mofetil and cyclophosphamide subgroups may be confounding.

Patients with RA have higher CV mortality than non-RA controls, independent of traditional CV risk factors, has been underestimated [30]. Moreover, hematuria, proteinuria, or CKD are associated with three- to four-fold higher mortality in patients with RA [3]. A recent study showed that in RA, renal dysfunction is associated with a higher risk of CV disease, independently of traditional CV risk factors [31]. However, the small number of CV events, confounding variables, and the lack of healthy control subjects limited the generalizability of these results [32].

### Study Strengths and Limitations

NHIRD data are generally accurate and reliable because the Taiwan NHI Bureau performs regular cross-checks and imposes heavy fines for false claims, overcharging, or malpractice. Earlier reports have affirmed the reliability of performing epidemiological research using the NHIRD LHID [33,34,35], and also that of the specific ICD-9-CM codes used to assess CKD outcomes [36,37,38].

By using a large national cohort with a longitudinal design and adjusting for several confounders, we have been able to affirm that CKD in RA patients is significantly associated with increased risk of ischemic heart disease and stroke, independent of traditional CV risk factors. Our findings concur with others which showed that CKD was independently associated with atherosclerosis and endothelial activation in RA, suggesting that endothelial dysfunction is central to the pathogenesis of CV complications in RA patients with CKD [39].

Our study had limitations. First, diagnoses of CKD, GN and ESRD were based entirely on secondary claim data, rather than eGFR. Thus, we were unable to stratify the risk of CKD in RA by CKD stage. However, others have documented the similarity of CKD diagnosis based on large administrative data sets and on eGFR [18,36]. Second, we have no renal biopsy pathology data. Third, because NHIRD lacks a reliable severity index for RA, we did not correlate disease activity with the prevalence of CKD. Although we were unable to adjust for potential unmeasured confounders, previous research suggests that such factors contribute far less to CKD than traditional risk factors, namely aging, diabetes, and hypertension [40], which were included in our analysis.

## Conclusions

This national cohort study demonstrates that the risks of CKD, GN and ESRD are significantly higher in patients with RA than the general population. The development of CKD in patients with RA is multifactorial and may result from several ongoing processes, including primary or secondary renal involvement associated with RA (eg, GN), chronic inflammation, comorbidities, and nephrotoxic antirheumatic drugs. Moreover, concurrent CKD disease predicts CV complications in RA patients, independent of other risk factors. Physicians should monitor the renal function of patients with RA regularly and intervene to tightly control CV risk factors and the progression of CKD, particularly in patients who are older, NSAID users, or have comorbidities.

## Supporting Information

S1 Table Frequency of glucocorticoids and NSAIDs use between patients with versus without RA. (DOCX)

S2 Table Distribution of age, comorbidity and medication between RA patients with frequent versus infrequent cyclosporine use. (DOCX)
