# Supplementary material for: Increased Risk of Chronic Kidney Disease in Rheumatoid Arthritis Associated with Cardiovascular Complications – A National Population-Based Cohort Study
Source: PLoS One. 2015 Sep 25;10(9):e0136508. doi: 10.1371/journal.pone.0136508 (PMC4583248; doi:10.1371/journal.pone.0136508)
Supplement: S1 Table — (DOCX) [file pone.0136508.s001.docx]

**Supporting Information**

**S1 Table**. Frequency of glucocorticoids and NSAIDs use between patients with versus without RA

|  | **Patients with RA, No. (%)** | **Patients without RA, No. (%)** | **P-value** |
| --- | --- | --- | --- |
| **Glucocorticoids** |  |  | <0.001 |
| Infrequent user^a^ | 4785/6055 (79%) | 9706/10639 (91%) |  |
| Frequent user^b^ | 1270/6055 (21%) | 923/10639 (9%) |  |
| **NSAIDs** |  |  | <0.001 |
| Infrequent user^a^ | 6122/12007 (51%) | 20963/28583 (73%) |  |
| Frequent user^b^ | 5885/12007 (49%) | 7620/28583 (27%) |  |

NSAID, non-steroidal anti-inflammatory drug; RA rheumatoid arthritis

^a^ Prescribed <90 days.

^b^ Prescribed ≥90 days.
