# Supplementary material for: Increased Risk of Chronic Kidney Disease in Rheumatoid Arthritis Associated with Cardiovascular Complications – A National Population-Based Cohort Study
Source: PLoS One. 2015 Sep 25;10(9):e0136508. doi: 10.1371/journal.pone.0136508 (PMC4583248; doi:10.1371/journal.pone.0136508)
Supplement: S2 Table — (DOCX) [file pone.0136508.s002.docx]

**Supporting Information**

**S2 Table .** Distribution of age, comorbidity and medication between RA patients with frequent versus infrequent cyclosporine use

|  | **Cyclosporine user, No. (%)** | |  |
| --- | --- | --- | --- |
| **Variable** | **Infrequency user (N=26)** | **Frequent user (N=73)** | **P-value** |
| **Age** (years) |  |  | 0.28 |
| 18–29 | 2 (7.7%) | 12 (16.4%) |  |
| 30–39 | 3 (11.5%) | 16 (21.9%) |  |
| 40–49 | 10 (38.5%) | 25 (34.2%) |  |
| 50–59 | 5 (19.2%) | 10 (13.7%) |  |
| 60–69 | 6 (23.1%) | 7 (9.6%) |  |
| ≥70 | 0 (0%) | 3 (4.1%) |  |
| **Comorbidity** |  |  |  |
| Hypertension | 9 (34.6%) | 22 (30.1%) | 0.67 |
| Diabetes | 6 (23.1%) | 9 (12.3%) | 0.20 |
| Cardiovascular disease | 8 (30.8%) | 15 (20.5%) | 0.29 |
| Hyperlipidemia | 4 (15.4%) | 11 (15.1%) | 0.97 |
| Obesity | 0 (0%) | 1 (1.4%) | 0.55 |
| **Drug** |  |  |  |
| Glucocorticoids | 24 (92.3%) | 70 (95.9%) | 0.47 |
| DMARDs | 26 (100%) | 73 (100%) | ND |
| NSAIDs | 25 (96.2%) | 73 (100%) | 0.09 |
| Etanercept | 3 (11.5%) | 14 (19.2%) | 0.38 |
| Adalimumab | 1 (3.8%) | 5 (6.8%) | 0.58 |

DMARD, disease-modifying antirheumatic drug; NSAID, non-steroidal anti-inflammatory drug; ND, not done; RA rheumatoid arthritis
